# Inferring Evolutionary Histories of Pathway Regulation from Transcriptional Profiling Data

**DOI:** 10.1371/journal.pcbi.1003255

**Published:** 2013-10-10

**Authors:** Joshua G. Schraiber, Yulia Mostovoy, Tiffany Y. Hsu, Rachel B. Brem

**Affiliations:** 1 Department of Integrative Biology, University of California, Berkeley, Berkeley, California, United States of America; 2 Department of Molecular and Cellular Biology, University of California, Berkeley, Berkeley, California, United States of America; EMBL-European Bioinformatics Institute & Wellcome Trust Sanger Institute, United Kingdom

## Abstract

One of the outstanding challenges in comparative genomics is to interpret the evolutionary importance of regulatory variation between species. Rigorous molecular evolution-based methods to infer evidence for natural selection from expression data are at a premium in the field, and to date, phylogenetic approaches have not been well-suited to address the question in the small sets of taxa profiled in standard surveys of gene expression. We have developed a strategy to infer evolutionary histories from expression profiles by analyzing suites of genes of common function. In a manner conceptually similar to molecular evolution models in which the evolutionary rates of DNA sequence at multiple loci follow a gamma distribution, we modeled expression of the genes of an *a priori*-defined pathway with rates drawn from an inverse gamma distribution. We then developed a fitting strategy to infer the parameters of this distribution from expression measurements, and to identify gene groups whose expression patterns were consistent with evolutionary constraint or rapid evolution in particular species. Simulations confirmed the power and accuracy of our inference method. As an experimental testbed for our approach, we generated and analyzed transcriptional profiles of four *Saccharomyces* yeasts. The results revealed pathways with signatures of constrained and accelerated regulatory evolution in individual yeasts and across the phylogeny, highlighting the prevalence of pathway-level expression change during the divergence of yeast species. We anticipate that our pathway-based phylogenetic approach will be of broad utility in the search to understand the evolutionary relevance of regulatory change.

## Author Summary

Comparative transcriptomic studies routinely identify thousands of genes differentially expressed between species. The central question in the field is whether and how such regulatory changes have been the product of natural selection. Can the signal of evolutionarily relevant expression divergence be detected amid the noise of changes resulting from genetic drift? Our work develops a theory of gene expression variation among a suite of genes that function together. We derive a formalism that relates empirical observations of expression of pathway genes in divergent species to the underlying strength of natural selection on expression output. We show that fitting this type of model to simulated data accurately recapitulates the parameters used to generate the simulation. We then make experimental measurements of gene expression in a panel of single-celled eukaryotic yeast species. To these data we apply our inference method, and identify pathways with striking evidence for accelerated or constrained regulatory evolution, in particular species and across the phylogeny. Our method provides a key advance over previous approaches in that it maximizes the power of rigorous molecular-evolution analysis of regulatory variation even when data are relatively sparse. As such, the theory and tools we have developed will likely find broad application in the field of comparative genomics.

## Introduction

Comparative studies of gene expression across species routinely detect regulatory variation at thousands of loci [1]. Whether and how these expression changes are of evolutionary relevance has become a central question in the field. In landmark cases, experimental dissection of model phenotypes has revealed evidence for adaptive regulatory change at individual genes [2]–[5]. These findings have motivated hypothesis-generating, genome-scale searches for signatures of natural selection on gene regulation. In addition to molecular-evolution analyses of regulatory sequence [6]–[9], phylogenetic methods have been developed to infer evidence for non-neutral evolutionary change from measurements of gene expression [10]–[12]. Two classic models of continuous character evolution have been used for the latter purpose: Brownian motion models, which can specify lineage-specific rates of evolution on a phylogenetic tree [13]–[16] and have been used to model the neutral evolution of gene expression [11], [17], and the Ornstein-Uhlenbeck model, which by describing lineage-specific forces of drift and stabilizing selection [13], [18], [19] can be used to test for evolutionary constraint on gene expression [11], [12]. To date, phylogenetic approaches have had relatively modest power to infer lineage-specific rates or selective optima of gene expression levels. This limitation is due in part to the sparse species coverage typical of transcriptomic surveys, in contrast to studies of organismal traits where observations in hundreds of species can be made to maximize the power of phylogenetic inference [20]–[22].

As a complement to model-based phylogenetic methods, more empirical approaches have also been proposed that detect expression patterns suggestive of non-neutral evolution [23]–[25]. We previously developed a paradigm to detect species changes in selective pressure on the regulation of a pathway, or suite of genes of common function, in the case where multiple independent variants drive expression of pathway genes in the same direction [24], [26]. Broadly, pathway-level analyses have the potential to uncover evidence for changes in selective pressure on a gene group in the aggregate, when the signal at any one gene may be too weak to emerge from genome-scale scans. However, the currently available tests for directional regulatory evolution are not well suited to cases in which some components of a pathway are activated, and others are down-regulated, in response to selection.

In this work, we set out to combine the rigor of phylogenetic methods to reconstruct histories of continuous-character evolution with the power of pathway-level analyses of regulatory change. We reasoned that an integration of these two families of methods could be used to detect cases of pathway regulatory evolution from gene expression data, without assuming a directional model. To this end, we aimed to develop a phylogenetic model of pathway regulatory change that accounted for differences in evolutionary rate between the individual genes of a pathway. We sought to use this model to uncover gene groups whose regulation has undergone accelerated evolution or been subject to evolutionary constraint, over and above the degree expected by drift during species divergence as estimated from genome sequence. As an experimental testbed for our inference strategy, we used the *Saccharomyces* yeasts. These microbial eukaryotes span an estimated 20 million years of divergence and have available well-established orthologous gene calls [27], and yeast pathways are well-annotated based on decades of characterization of the model organism *S. cerevisiae*. We generated a comparative transcriptomic data set across Saccharomycetes by RNA-seq, and we used the data to search for cases of pathway regulatory change.

## Results

### Modeling the rates of regulatory evolution across the genes of a pathway

The Brownian-motion model of expression of a gene predicts a multivariate normal distribution of observed expression levels in the species at the tips of a phylogenetic tree. The variance-covariance matrix of this multivariate normal distribution reflects both the relatedness of the species and the rate of regulatory evolution along each branch of the tree. We sought to apply this model to interpret expression changes in a pre-defined set of genes of common function, which we term a pathway. Our goal was to test for accelerated or constrained regulatory variation in a pathway relative to the expectation from DNA sequence divergence, as specified by a genome tree. To avoid the potential for over-parameterization if the rate of each gene in a pathway were fit separately, we instead developed a formalism, detailed in Methods, to model regulatory evolution in the pathway using a parametric distribution of evolutionary rates across the genes. This strategy parallels well-established models of the rate of DNA sequence evolution across different sites in a locus or genome [28]. Briefly, we assumed that each gene in the pathway draws its rate of evolution from an inverse gamma distribution, and we derived the relationship between the parameters of this distribution and the likelihood of expression observations at the tips of the tree. For each gene, we modeled the contrasts of the expression level in each species relative to an arbitrary species used as a reference, to eliminate the need to estimate the ancestral expression level. A further normalization step, recentering the distribution of expression across pathway genes in each species to a mean of 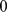, corrected for the effects of coherent regulatory divergence due to drift. This formalism enabled a maximum-likelihood fit of the parameters describing the pathway expression distribution, given empirical expression data, and could accommodate models of lineage-specific regulatory evolution, in which a particular subtree was described by distinct evolutionary rate parameters relative to the rest of the phylogeny. As a point of comparison, we additionally made use of an Ornstein-Uhlenbeck (OU) model [19]: here the rate of regulatory evolution of each gene in a pathway, across the entire phylogeny, was drawn from an inverse-gamma distribution, and all genes of the pathway were subject to the same degree of stabilizing selection, again across the entire tree.

Our ultimate application of the method given a set of expression data was to enumerate all possible Brownian motion models in which pathway expression evolved at a distinct rate along the lineages of a subtree relative to the rest of the phylogeny, and for each such model, apply our fitting strategy and tabulate the likelihood of the data under the best-fit parameter set. To compare these likelihoods and the analogous likelihood from the best-fit OU model of universal constraint, we applied a standard Akaike information criterion (AIC) [21], [29], [30] to identify strongly supported models.

### Simulation testing of inference of pathway regulatory evolution

**Figure 1:**
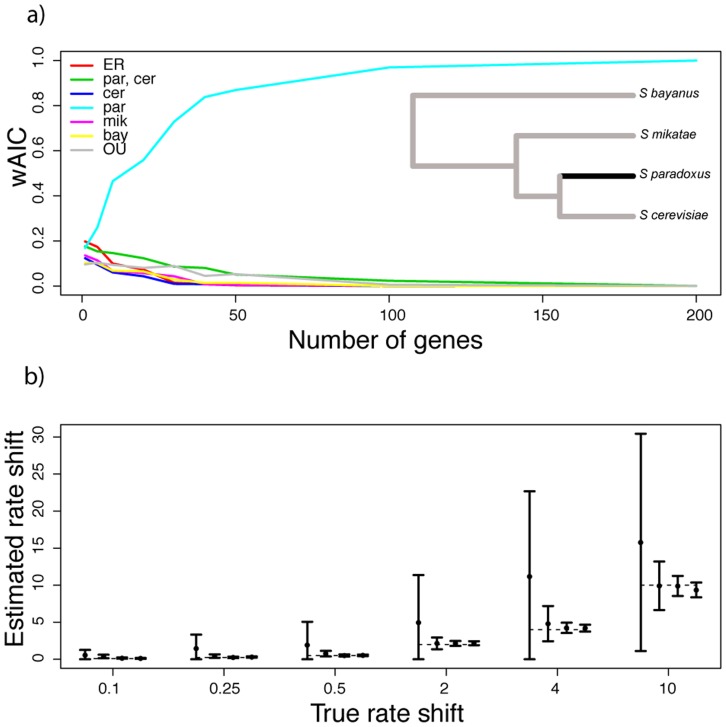
Phylogenetic inference of the evolutionary history of yeast pathway regulation from data simulated under a model of a lineage-specific, accelerated evolutionary rate. Each panel reports results of the inference of evolutionary history from expression of the genes of a pathway in yeast species, simulated under a model of a shift in evolutionary rate on the branch leading to *S. paradoxus* (dark line in inset phylogeny in (a)). (a), Each trace reports the strength of support for one evolutionary model in inferences from simulated expression in pathways of varying size. The 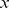 axis reports the number of genes in the pathway and the 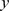 axis reports the Akaike weight of the indicated model. Data were simulated under a Brownian motion model in which the rate of regulatory evolution for each gene was drawn from an inverse-gamma distribution with 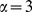, 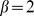 and, for the branch leading to *S. paradoxus*, increased by a factor of 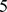. In the legend, ER denotes an equal-rates Brownian motion model in which rates of evolution were the same on each branch of the phylogeny; OU denotes an Ornstein-Uhlenbeck model of evolution; and species name abbreviations denote Brownian motion models of accelerated evolutionary rate on the subtrees leading to the respective taxa. (b), Each set of symbols reports results from expression data simulated under a Brownian motion model in which the rate of regulatory evolution for each gene was drawn from an inverse-gamma distribution with 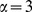, 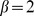 and, for the branch leading to *S. paradoxus*, increased by the factor indicated on the 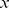 axis. In a given set of symbols, filled circles report the mean, and vertical bars report the standard deviation of the sampling distribution, of the inferred rate shift parameter in simulations of pathways containing, from left to right, 2, 10, 50, and 100 genes. Results from simulations of expression under models of evolutionary rate shifts on other branches of the yeast phylogeny, and simulations of expression in the absence of a lineage-specific evolutionary rate shift, are reported in Supplmentary Figures 1–5.

As an initial test of our approach, we sought to assess the performance of our phylogenetic inference scheme in the ideal case in which rates of regulatory evolution of the genes of a pathway were simulated from, and thus conformed to, the models of our theoretical treatment. In keeping with our experimental application below which used a comparison of *Saccharomyces* yeast species as a testbed, we developed a simulation scheme using a molecular clock-calibrated *Saccharomyces* phylogeny [27] (see Figure 1a inset). We simulated the expression of a multi-gene pathway in which rates of evolution of the member genes were drawn from an inverse gamma distribution. With the simulated expression data in hand from a given generating model, we fit an OU model, an equal-rates model, and models of evolutionary rate shifts in each subtree in turn.

**Figure 2:**
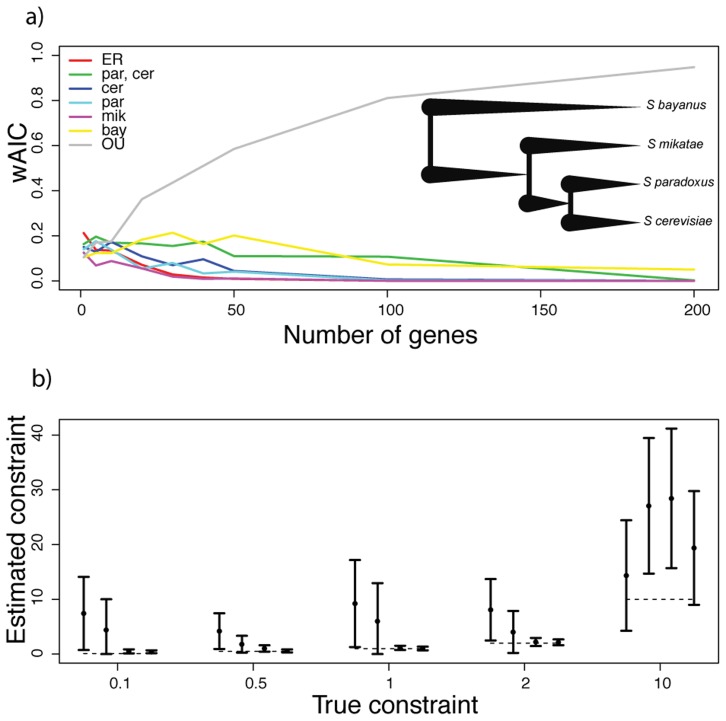
Phylogenetic inference of the evolutionary history of yeast pathway regulation from data simulated under an Ornstein-Uhlenbeck (OU) model. (a), Data are as in Figure 1a except that expression measurements were simulated under an OU model in which the phylogeny-wide rate of regulatory evolution for each gene was drawn from an inverse-gamma distribution with 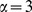, 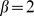 and the phylogeny-wide constraint parameter had a value of 10. (b), Data are as in Figure 1b except that expression measurements were simulated under an OU model in which the phylogeny-wide rate of regulatory evolution for each gene was drawn from an inverse-gamma distribution with 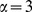, 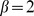 and the phylogeny-wide constraint parameter had the value indicated on the 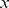 axis.

Figure 1 shows the results of inferring the mode and rate of evolution from data simulated under a model of accelerated regulatory change on the branch leading to *S. paradoxus*, and similar results can be seen in Figures S1 through S5 for other rate shift models. As expected, for very small gene groups, inference efforts did not achieve high power or recapitulate model parameters (Figure 1a, leftmost data point; Figure 1b, leftmost point in each cluster), reflecting the challenges of the phylogenetic approach when applied on a gene-by-gene basis to relatively sparse trees like the *Saccharomyces* species set. By contrast, for pathways of ten genes or more, we observed strong AIC support for the true generating model in cases of lineage-specific regulatory evolution, approaching AIC weights of 100% for the correct model if a pathway contained more than 50 genes (Figure 1a, Figure S1 and panel a of Figures S2, S3, S4, S5). In these simulations our method also inferred the correct magnitudes of lineage-specific shifts with high confidence, for all but the smallest pathways (Figure 1b and panel b of Figures S2, S3, S4, S5). Likewise, when applied to simulated expression data generated under models of phylogeny-wide constraint, our method successfully identified OU as the correct model (Figure 2a), though with biased estimates of the magnitude of the constraint parameter when the latter was large (Figure 2b), likely due to a lack of identifiability with the inverse-gamma rate parameter (Figure S6).

We also sought to evaluate the robustness of our method to violations of the underlying model. To explore the effect of our assumption of independence between genes, we simulated a pathway in which expression of the individual genes was coupled to one another and evolving under an equal-rates Brownian motion model, and we inferred evolutionary histories either including or eliminating the mean-centering normalization step of our analysis pipeline. With the latter step in place, our method correctly yielded little support for shifts in evolutionary rates in the simulated data except in the case of extremely tight correlation between genes, a regime unlikely to be biologically relevant (Figure S7). Additionally, to test the impact of our assumption that the genes of a pathway were all subject to similar evolutionary pressures, we simulated a heterogeneous pathway in which expression of only a fraction of the gene members was subject to a lineage-specific shift in evolutionary rate. Inferring parameters from these data revealed accurate detection of rate shifts even when a large proportion of the genes in the pathway deviated from the rate shift model (Figure S8). Taken together, our results make clear that the pathway-based phylogenetic approach is highly powered to infer evolutionary histories of gene expression change, particularly lineage-specific evolutionary rate shifts. As a contrast to the poor performance of phylogenetic inference when applied to one or a few genes, our findings underscore the utility of the multi-gene paradigm in identifying candidate cases of evolutionarily relevant expression divergence.

### Phylogenetic inference of regulatory evolution from experimental measurements of *Saccharomyces* expression

We next set out to apply our method for evolutionary reconstruction of regulatory change to experimental measurements of gene expression. The total difference in gene expression between any two species is a consequence of heritable differences that act in *cis* on the DNA strand of a gene whose expression is measured, and of variants that act in *trans*, through a soluble factor, to impact gene expression of distal targets. Effects of *cis*-acting variation can be surveyed on a genomic scale using our previously reported strategy of mapping of RNA-seq reads to the individual alleles of a given gene in a diploid inter-specific hybrid [24], whereas the joint effects of *cis* and *trans*-acting factors can be assessed with standard transcriptional profiling approaches in cultures of purebred species. To apply these experimental paradigms we chose a system of *Saccharomyces sensu stricto* yeasts. We cultured two biological replicates for each of a series of hybrids formed by the mating of *S. cerevisiae* to *S. paradoxus*, *S. mikatae*, and *S. bayanus* in turn, as well as homozygotes of each species. We measured total expression in the species homozygotes, and allele-specific expression in the hybrids, of each gene by RNA-seq, using established mapping and normalization procedures, including a variance-stabilizing full-quantile normalization (see Methods). In each set of expression data, we made use of *S. cerevisiae* as a reference: we normalized expression in the homozygote of a given species, and expression of the allele of a given species in a diploid hybrid, relative to the analogous measurement from *S. cerevisiae*.

**Figure 3:**
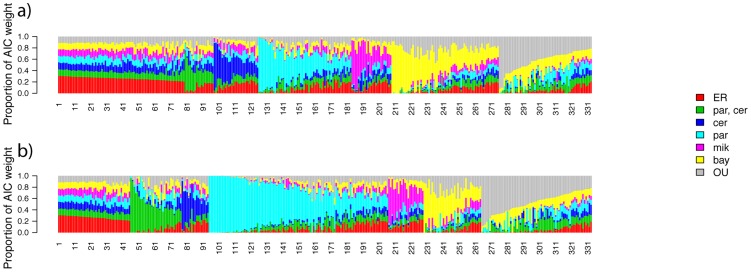
Inference of regulatory evolution in yeast pathways from experimental expression measurements. Each panel reports results of phylogenetic inference of evolutionary histories of gene expression change from one set of experimental transcriptional profiling data. In a given panel, each vertical bar reports results of maximum-likelihood fits of Brownian-motion and Ornstein-Uhlenbeck models to expression of the genes of one Gene Ontology process term; the total proportion of a bar corresponding to a particular color indicates the Akaike weight of the corresponding model (legend at right, with labels as in Figure 1). Bars are sorted by the model with maximum Akaike weight. (a), Inference of *cis*-regulatory variation from interspecies hybrids; numerical indices correspond to rows in Table S3. (b), Inference from measurements of total expression in species homozygotes; numerical indices correspond to rows in Table S4.

To search for evidence of evolutionary constraint and lineage-specific shifts in evolutionary rate in our yeast expression data, we considered as pathways the pre-defined sets of genes of common function from the Gene Ontology (GO) process categories. For the genes of each GO term, we used normalized expression measurements in yeast species and, separately, measurements of *cis*-regulatory variation from interspecific hybrids, as input into our phylogenetic analysis pipeline. Thus, for each of the two classes of expression measurements, for a given GO term we fit models of a lineage-specific rate shift in regulatory evolution incorporating inverse-gamma-distributed rates across genes; an analogous model with no lineage-specific rate shift; and an OU model of universal constraint. The results revealed a range of inferred evolutionary models and AIC support across GO terms (Figure 3, Tables 1 and 2, and Tables S3 and S4), and this complete data set served as the basis for manual inspection of biologically interesting features.

**Table 1:** Top-scoring fitted models of *cis*-regulatory evolution in yeast pathways from experimental expression measurements.

GO term	*N*	Model	wAIC	Constraint or shift parameter
34599	57	Ornstein-Uhlenbeck	0.899405768	49.97745883
6355	433	*S. bayanus* shift	0.837382338	0.230918849
6351	462	*S. bayanus* shift	0.849912647	0.258701476
1302	38	*S. paradoxus* shift	0.859866949	3.197059161
6897	73	*S. paradoxus* shift	0.965743399	4.292287639
6338	45	*S. cerevisiae* shift	0.840339574	0.037806902
42254	136	Ornstein-Uhlenbeck	0.924785133	3.733770466
6364	177	Ornstein-Uhlenbeck	0.902358815	3.079387696
44255	13	*S. paradoxus* shift	0.945799302	11.43989834
54	11	*S. paradoxus* shift	0.91523272	9.314688245
16310	188	*S. bayanus* shift	0.902247359	0.188381056
8152	243	*S. bayanus* shift	0.844716856	0.043114988
6629	136	*S. bayanus* shift	0.91650274	0.005082617
122	71	*S. bayanus* shift	0.819216472	0.040060263
30437	45	*S. paradoxus* shift	0.931136455	4.060128813

Each row reports the results of phylogenetic inference of the evolutionary history of gene regulation for one yeast Gene Ontology process term, from experimental measurements of *cis*-regulatory variation in interspecific yeast hybrids. 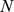, number of genes in the indicated GO term for which expression measurements were available in all species. Model, best-fit model from among the five possible Brownian motion models of evolutionary rate shift in lineages of the *Saccharomyces* phylogeny (see Figure 1a), the Ornstein-Uhlenbeck (OU) model of universal constraint, and the equal-rates model involving no lineage-specific differences in evolutionary rate. wAIC, Akaike Information Criterion weight of the indicated model. Constraint or shift parameter, fitted value of the strength of purifying selection or the shift in the rate of regulatory evolution on the indicated lineage, when the best-fit model was the OU model of constraint or a Brownian motion lineage-specific evolutionary rate model, respectively.

**Table 2:** Top-scoring fitted models of species regulatory evolution in yeast pathways from experimental expression measurements.

GO term	*N*	Model	wAIC	Constraint or shift parameter
6397	151	*S. paradoxus* shift	0.965171603	3.028130303
8033	69	*S. paradoxus* shift	0.969683391	3.714749932
71038	15	*S. paradoxus* shift	0.89725301	6.751073973
480	29	*S. paradoxus* shift	0.928296518	4.460579672
42274	25	*S. paradoxus* shift	0.958076119	8.083546161
472	31	*S. paradoxus* shift	0.953733629	4.686648741
15031	362	*S. bayanus* shift	0.872939854	0.183834463
1302	38	*S. paradoxus* shift	0.999927135	6.671016575
6006	22	*S. paradoxus* shift	0.816341854	4.6555377
6260	72	*S. paradoxus* shift	0.831407464	3.043207869
30163	15	*S. paradoxus* shift	0.82364567	7.009201233
6897	73	*S. paradoxus* shift	0.970677101	4.408614609
6412	228	*S. paradoxus* shift	0.981277345	2.770778823
7121	16	*S. paradoxus* shift	0.998579562	16.81960721
6914	49	Ornstein-Uhlenbeck	0.810293525	41.38598192
30488	18	*S. paradoxus* shift	0.893282646	7.945094861
42254	163	*S. paradoxus* shift	0.99999983	6.856141937
6200	34	*S. paradoxus* shift	0.81144199	5.590943868
6468	120	*S. paradoxus* shift	0.990399439	2.655209273
16567	71	*S. paradoxus* shift	0.959694914	3.313920599
6364	177	*S. paradoxus* shift	0.999995709	5.841035759
6754	18	*S. paradoxus* shift	0.816303046	4.668929462
422	27	Ornstein-Uhlenbeck	0.877576591	57.08946364
463	20	*S. paradoxus* and *S. cerevisiae* shift	0.958484282	10.39289039
6414	23	*S. paradoxus* and *S. cerevisiae* shift	0.906687775	8.121469425
19236	29	*S. paradoxus* shift	0.989881765	6.821984459
31505	72	*S. paradoxus* shift	0.955855579	3.032267535
32259	65	*S. paradoxus* shift	0.998665437	4.546902844
6506	29	*S. paradoxus* shift	0.982054204	5.468542886
16310	188	*S. paradoxus* shift	0.99652632	2.487101867
447	39	*S. paradoxus* shift	0.994506418	5.252074336
6281	175	Ornstein-Uhlenbeck	0.882367142	3.410968446
71042	13	*S. paradoxus* shift	0.804318406	6.030946867
6378	18	*S. cerevisiae* shift	0.845112064	1.00E-04
7165	63	*S. paradoxus* shift	0.811091269	4.465389345
6810	681	Ornstein-Uhlenbeck	0.859937275	2.618523967
6812	28	*S. paradoxus* shift	0.898839416	4.312524185
8150	723	*S. paradoxus* shift	0.999962114	2.871955612
6417	45	*S. paradoxus* shift	0.925463092	5.339113187
6407	18	*S. paradoxus* shift	0.988260506	8.792447836
462	55	*S. paradoxus* shift	0.817627126	7.291083934

Data are as in Table 1 except that inferences were made from experimental measurements of expression in purebred yeast homozygotes.

**Figure 4:**
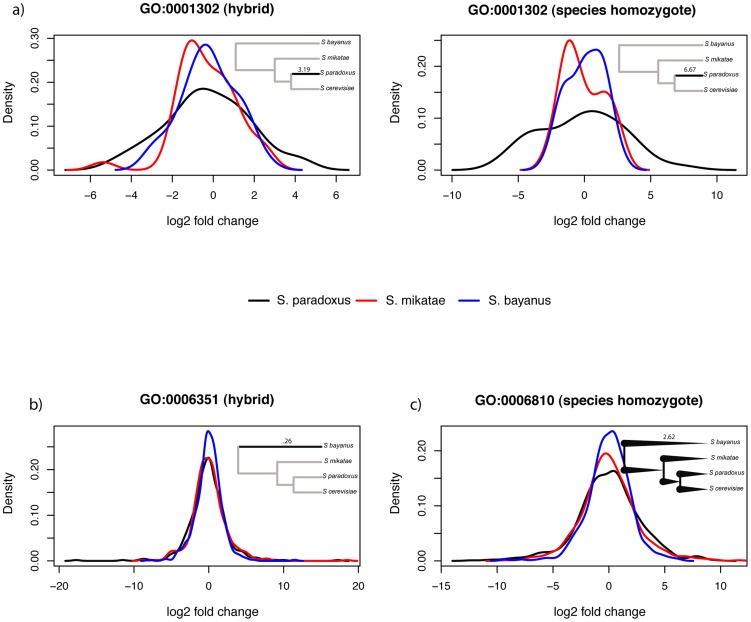
Lineage-specific regulatory evolution and constraint in yeast pathways, inferred from experimental expression measurements. Each panel shows kernel density estimates of the distributions of experimental gene expression measurements among the genes of one yeast Gene Ontology process term, whose evolutionary history was inferred with strong support. In a given panel, each trace reports the expression levels of the genes of the indicated pathway, from the allele of the indicated yeast species in a hybrid or in the purebred homozygote of a species, normalized with respect to the analogous measurement in *S. cerevisiae* and with respect to branch length. Inset cartoons represent the model inferred with AIC weight >80% for the indicated pathway (see Tables 1 and 2). (a) Allele-specific expression from measurements in diploid hybrids (left) and total expression measurements in species homozygotes (right) for the 38 genes of GO:0001302, replicative cell aging, supporting a model of accelerated evolution in *S. paradoxus*; in the inset, the number above the bolded branch reports the inferred shift in the rate of regulatory evolution along that lineage. (b) Allele-specific expression from measurements in diploid hybrids for the 462 genes of GO:0006351, transport, supporting a model of constraint in *S. bayanus*; in the inset, the number above the bolded branch reports the inferred shift in the rate of regulatory evolution along that lineage. (c) Total expression measured in species homozygotes for the 175 genes of GO:0006281, transcription, supporting an Ornstein-Uhlenbeck model of universal constraint; in the inset, the number above the tree reports the inferred value of the constraint parameter. Note that in (c), the width of the distribution of expression differences between a given species and *S. cerevisiae* correlates inversely with the sequence divergence of that species, as expected if selective constraint on expression renders the estimate of evolutionary distance from genome sequence an increasing over-estimate of expression change.

Among the inferences of pathway regulatory evolution from our method, we observed many cases of evolutionary interest whose best-fitting model had strong AIC support (Figure 3). For each of 15 GO terms, *cis*-regulatory expression variation measurements yielded inference of an evolutionary model with >80% AIC weight (Figure 3a and Table 1). Many such GO terms represented candidate cases of polygenic regulatory evolution, in which multiple independent variants, at the unlinked genes that make up a pathway, have been maintained in some yeast species in response to a lineage-specific shift in selective pressure on expression of the pathway components. For example, in replicative cell aging genes (GO term 0001302), *cis*-regulatory variation measured in interspecific hybrids supported a model of polygenic, accelerated evolution in *S. paradoxus* (Figure 4a), with some pathway components upregulated and some downregulated in the latter species relative to other yeasts. The total expression levels of cell aging genes in species homozygotes were also consistent with rapid evolution in *S. paradoxus* (Figure 4a), arguing against a model of compensation between *cis*- and *trans*-acting regulatory variation, and highlighting this pathway as a particularly compelling potential case of a lineage-specific change in selective pressure.

In other instances, expression measurements in species homozygotes alone supported models of lineage-specific evolution, with each such pathway representing a candidate case of accelerated or constrained evolution at *trans*-acting regulatory factors. For a total of 41 GO terms, our method inferred models with >80% AIC weight from homozygote species expression data (Figure 3b and Table 2). These top-scoring pathways included a set of components of the transcription machinery (GO term 0006351), whose expression levels in *S. bayanus* were less volatile than those of other yeasts and thus supported a model of lineage-specific constraint (Figure 4b). Additionally, expression of a number of pathways in species homozygotes conformed to the OU model of universal constraint, such as a set of genes annotated in transport (GO term 0006281), whose expression varied less across all species than would be expected from the genome tree (Figure 4c). Taken together, our findings indicate that evolutionary histories can be inferred with high confidence from experimental measurements of pathway gene expression. In our yeast data, many pathways exhibit expression signatures consistent with non-neutral regulatory evolution, in particular lineages and across the phylogeny.

Another emergent trend was the prevalence, across many GO terms, of models of distinct regulatory evolution in the lineage to *S. paradoxus* as the best fit to expression measurements in species homozygotes (Figure 3b). We noted no such recurrent model in analyses of *cis*-regulatory variation (Figure 3a), implicating *trans*-acting variants as the likely source of the regulatory divergence in *S. paradoxus*. To validate these patterns, we applied our phylogenetic inference method to expression measurements from all genes in the genome analyzed as a single group, rather than to each GO term in turn. When we used expression data from species homozygotes as input for this genome-scale analysis, our method assigned complete AIC support to a model in which the rate of evolution was 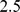 times faster on the branch leading to *S. paradoxus* (AIC weight 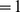), consistent with results from individual GO terms (Figure 3b). An analogous inference calculation using measurements of *cis*-regulatory variation, for all genes in the genome, yielded essentially complete support for an OU model of universal constraint (AIC weight 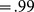). We conclude that constraint on the *cis*-acting determinants of gene expression, of roughly the same degree in all yeasts, is the general rule from which changes in selective pressure on particular functions may drive deviations in individual pathways. However, for many genes, expression in the *S. paradoxus* homozygote is distinct from that of other yeasts out of proportion to its sequence divergence, suggestive of derived, *trans*-acting regulatory variants with pleotropic effects.

## Discussion

The effort to infer evolutionary histories of gene expression change has been a central focus of modern comparative genomics. Against a backdrop of a few landmark successes [11], [12], progress in the field has been limited by the relatively weak power of phylogenetic methods when applied, on a gene-by-gene basis, to measurements from small sets of species. In this work, we have met this challenge with a method to infer evolutionary rates of any suite of independently measured continuous characters that can be analyzed together across species. We have derived the mathematical formalism for this model, and we have illustrated the power and accuracy of our approach in simulations. We have generated yeast transcriptional profiles that complement available data sets [31], [32] by measuring *cis*-regulatory contributions to species expression differences as well as the total variation between species. With these data, we have demonstrated that our phylogenetic inference method yields robust, interpretable candidate cases of pathway regulatory evolution from experimental measurements.

The defining feature of our phylogenetic inference method is that it gains power by jointly leveraging expression measurements of a group of genes, while avoiding a high-dimensional evolutionary model. Rather than requiring an estimate of the evolutionary rate at each gene, our strategy estimates the parameters of a distribution of evolutionary rates across genes. We thus apply the assumption of [10] and model expression of the individual genes of a pathway as independent draws from the same distribution, mirroring the standard assumption of independence across sites in phylogenetic analyses of DNA sequence [33]. Any observation of lineage-specific *cis*-acting regulatory variation from our approach is of immediate evolutionary interest: a species-specific excess of variants at unlinked loci of common function would be unlikely under neutrality, and would represent a potential signature of positive selection if fixed across individuals of the species. In the study of *trans*-acting regulatory variation, *a priori* a case of apparent accelerated evolution of a pathway could be driven by a single mutation of large effect maintained by drift in a species, as in any phenomenological analysis of trait evolution [13], [34]. Our results indicate that for correlated gene groups, the latter issue can be largely resolved by a simple transformation in which expression of each gene is normalized against the mean of all genes in the pathway. Additional corrections could be required under more complex models of correlation among pathway genes, potentially to be incorporated with matrix-regularization techniques that highlight patterns of correlation in transcriptome data [35]. Similarly, although the assumption of independence across genes could upwardly bias the likelihoods of best-fit models in our inferences, model choice and parameter estimates will still be correct on average even with the scheme implemented here [36].

Our strategy also assumes that the genes of a pre-defined pathway are subject to similar evolutionary pressures. Simulation results indicate that this assumption does not compromise the performance of our method, as we observed robust inference to be the rule rather than the exception even in a quite heterogeneous pathway, if a proportion of the genes evolved under a rate shift model. Although we have used pathways defined by Gene Ontology in this study, our method can easily be applied to gene modules defined on the basis of protein or genetic interactions or coexpression. Any such module is likely to contain both activators and repressors, or other classes of gene function whose expression may be quantitatively tuned in response to selection by alleles with effects of opposite sign [37], [38]. The phylogenetic approach we have developed here is well-suited to detect these non-directional regulatory patterns, rather than relying on the coherence of up- or down-regulation of pathway genes [24]–[26], [39]–[41]. Ultimately, a given case of strong signal in our pathway evolution paradigm, when the best-fit model is one of lineage-specific accelerated regulatory evolution, can be explained either as a product of relaxed purifying selection or positive selection on pathway output. Our approach thus serves as a powerful strategy to identify candidates for population-genetic [26] and empirical [40], [42] tests of the adaptive importance of pathway regulatory change. We have developed an R package, PIGShift (Polygenic Inverse Gamma rateShift), to facilitate the usage of our method. The pathway-level approach is not contingent on the Gaussian models of regulatory evolution we have used here, and future work will evaluate the advantages of compound Poisson process [10], [43] or more general Lévy process [44] models of gene expression, as well as models that account explicitly for sampling error in expression data.

The advent of RNA-seq has enabled expression surveys across non-model species in many taxa. Maximizing the biological value of these data requires methods that evaluate expression variation in the context of sequence divergence between species. As rigorous phylogenetic interpretation of expression data becomes possible, these measurements will take their place beside genome sequences as a rich source of hypotheses, in the search for the molecular basis of evolutionary novelty.

## Methods

### Basic model

Our basic assumption, following [10], is that the average expression levels of genes in a pathway evolve as independent replicates of the same Brownian motion or Ornstein-Uhlenbeck process. However, instead of assuming that each gene in the pathway has the same rate of evolution, we allow the different genes in a pathway to draw their rate of evolution from a parametric distribution.

As a point of departure, we begin by considering the likelihood of a group of genes whose expression evolves independently, each with its own rate of evolution. Throughout, we use uppercase letters to represent random variables and matrices and lowercase letters to represent nonrandom variables. Assume that we have measured expression of the genes of a pathway in 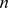 species, and that we have a fixed, time-calibrated phylogeny from genome sequence data describing the relationships between those species. We let 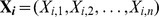 be the observations of the expression level of the 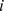
th gene of the pathway, in each of 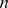 species. Both the Brownian- motion and Ornstein-Uhlenbeck (OU) models predict that the vector 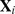 is a draw from a multivariate normal distribution with variance-covariance matrix 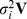 (where 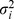 is a scalar—the rate of evolution—and the elements of 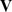 depend on whether evolution follows the Brownian or Ornstein-Uhlenbeck model; see below). Hence, the likelihood of the data is
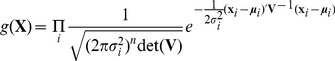
(1)where 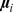 is a vector representing the mean expression value at the tips of the phylogenetic tree for gene 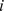. Note that 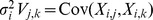 where 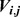 is the 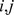
th element of 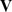.

If we assume that there is no branch-specific directionality to evolution, we can avoid the need to estimate 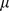 in either the Brownian motion model or the OU model by a renormalization of the data. We first arbitrarily choose the gene expression measurements in a single species (say species 1), and define the new random vector 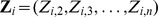 by
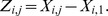
By our assumption that there is no branch-specific directionality, 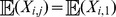 so 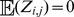 for all 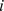 and 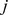. Because each 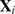 is multivariate normally distributed with dimension 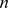, each 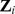 will also be multivariate normally distributed with dimension 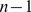 and a slightly different covariance structure. Letting 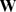 be the covariance matrix corresponding to the 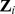, elementary calculations taking into account variances and covariances of sums of random variables reveal that
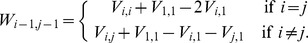
Next, we wish to incorporate into the Brownian motion and OU models a scheme in which the rates of evolution of the genes of a pathway are not specified independently but instead are drawn from an inverse-gamma distribution. In this context, the genes in a pathway share 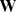, the variance-covariance structure due to the tree, but the rate of evolution 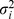 for each gene is an independent draw from an inverse-gamma distribution. The inverse-gamma distribution has density
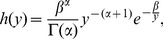
(2)where 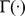 is the gamma function and 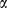 and 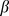 are shape and scale parameters. The moments of this distribution are
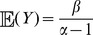
and
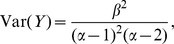
from which it follows that the inverse-gamma distribution has no mean if 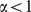 and no variance if 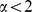. These properties allow for the distribution of rates of gene expression evolution in a pathway to be relatively broad; in addition, the inverse gamma density has no mass at 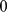, which prevents any gene in a pathway from not evolving at all. Also, as 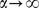 and 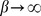 as 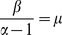 stays fixed, the distribution converges to a point mass at 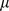. Thus, a model where there is one rate for every gene is nested within the inverse-gamma distributed rates model.

Computation of the the likelihood of the data under this model is simplified by the fact that the inverse-gamma distribution is the conjugate prior to the variance of a normal distribution. Hence, we see that the likelihood of the observed expression data 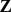 is
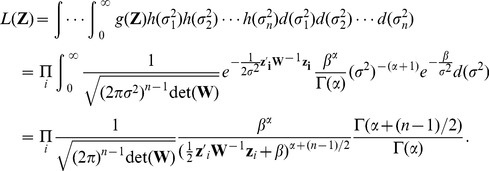
(3)


The second line follows recognizing that each integral is independent. Thus, the likelihood of the observations of transcriptome-wide gene expression across the pathway in 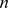 taxa, normalized by the expression level in taxon 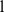, is given by (3).

For the application to simulated and experimental data as described below, given observations of gene expression of the species at the tips of the tree, and a model that specifies the covariance matrix 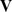 detailed in the next section, we optimized the log likelihood function using the L-BFGS-B optimization routine in R [45].

### Covariance matrix

In the previous section, we left the unnormalized covariance matrix 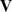 unspecified. Here we briefly recall the forms of 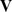 under Brownian motion and the Ornstein-Uhlenbeck process. Define the height of the evolutionary tree to be 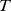 and and the height of the node containing the common ancestor of taxa 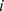 and 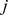 by 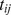. Then the covariance matrix for Brownian motion is
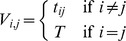
and the covariance matrix for the Ornstein-Uhlenbeck process is
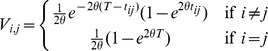
where 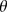 quantifies the strength of stabilizing selection, with large 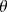 corresponding to stronger selection.

To model lineage-specific shifts in the evolutionary rate of gene expression in the context of the Brownian motion model, we adopt a framework similar to that of O'Meara *et al.* [15]. We assume that in a specified subtree of the total phylogeny, the rate of evolution of every gene is multiplied by a constant, compared to the rest of the tree. Under the Brownian motion model, this is equivalent to multiplying the branch lengths in that part of the tree by that same constant; hence, shifts in evolutionary rate are incorporated by multiplying the branch lengths of affected branches by the value of the rate shift.

### Comparing likelihoods among fitted models

To evaluate the support for the distinct models we fit to expression data for a given pathway, we require a strategy that will be broadly applicable in cases where no *a priori* expectation of the correct model is available, such that nested hypothesis testing schemes [15] are not applicable. Instead, given likelihoods 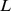 from fitting of each model in turn to expression data from the genes of a pathway, we use the Akaike Information Criterion, 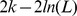 [46], to report the strength of the support for each, where 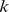 is the number of parameters in the model (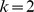 for the Brownian motion model in which the rate of evolution is the same along all lineages in the phylogeny, and 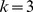 for all other models).

### Simulations

For all simulations, we used a phylogenetic tree adapted from [27] by removing the branch leading to *Saccharomyces kudriavzevii* (see inset of Figure 1a and Figures S1, S2, S3, S4, S5). To simulate under models in which each gene in a pathway evolves independently, we generated expression data for one gene at a time as follows. We first drew the rate of evolution from the appropriately parameterized inverse-gamma distribution. Then, without loss of generality, we specified that the expression level at the root of the phylogeny was equal to 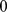, and we simulated evolution along the branches of the yeast phylogeny according to either a Brownian motion or an Ornstein-Uhlenbeck process (with optimal expression level equal to 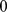), using the terminal expression level on a branch as the initial expression level of its daughter branches. To account for lineage-specific shifts in evolutionary rate in a simulated pathway, we multiplied the rate of evolution of each gene by the rate shift parameter for evolution along the branches affected by the rate shift. For each Brownian motion-based rate shift model applicable to the tree, we simulated 100 replicate datasets for each of a range of gene group sizes, in each case setting 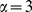, 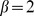, and the rate shift parameter as specified in Figure 1 and Figures S1, S2, S3, S4, S5. For the Ornstein-Uhlenbeck model, we simulated 100 replicate datasets for each of a range of pathway sizes with 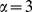, 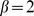, and 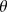 as specified in Figure 2.

To simulate under models in which expression of genes in a pathway was correlated with coefficient 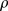, we first drew 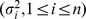, the rate of evolution for each gene, from an inverse-gamma distribution with 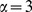, 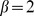. We then parameterized the instantaneous variance-covariance matrix of the 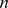-dimensional Brownian motion by
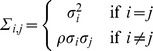
so that the distribution of trait change along a lineage was multivariate normal with mean 0 and variance covariance matrix 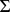. Separate simulated expression data sets were generated with 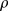 varying from 0 (complete independence) to 1 (complete dependence) using 100 replicate simulations for each value.

### Yeast strains, growth conditions, and RNA-seq

Strains used in this study are listed in Table S1. For pairwise comparisons of *S. cerevisiae* and each of *S. paradoxus*, *S. mikatae*, and *S. bayanus*, two biological replicates of each diploid parent species and each interspecific hybrid were grown at 25°C in YPD medium [47] to log phase (between 0.65–0.75 OD at 600 nm). Total RNA was isolated by the hot acid phenol method [47] and treated with Turbo DNA-free (Ambion) according to the manufacturer's instructions. Libraries for a strand-specific RNA-seq protocol on the Illumina sequencing platform, which delineates transcript boundaries by sequencing poly-adenylated transcript ends, were generated as in [48] with the following modifications: 1) AmpureXP beads (Beckman) were used to clean up enzymatic reactions; 2) the gel purification and size-selection step was eliminated; 3) the oligo-dT primer used for cDNA synthesis was phosphorothioated at position ten (TTTTTTTTTT*TTTTTTTTTTVN, V = A,C,G, N = A,C,G,T, * = phosphorothioate linkage, Integrated DNA Technologies); and 4) 12 PCR cycles were performed. Libraries were sequenced using 36 bp paired-end modules on an Illumina IIx Genome Analyzer (Elim Biopharmaceuticals).

### RNA-seq mapping and normalization

Bioinformatic analyses were conducted in Python and R. RNA-seq reads were stripped of their putative poly-A tails by removing stretches of consecutive Ts flanking the sequenced fragment; reads without at least two such Ts were discarded, as were reads with Ts at both ends. To ensure that expression data from hybrid diploids and purebred species could be compared, for each class of expression measurement for a given pair of species we mapped reads to both species genomes from http://www.saccharomycessensustricto.org [27] using Bowtie [49] with default settings and flags -m1 -×1000. These settings allowed us to retain only those reads that were unambiguously assigned to one of the two species in each pairwise comparison. A mapped read was inferred to have originated from the plus strand of the genome if its poly-A tail corresponded to a stretch of As at the 3′ end of the fragment, and a read was assigned to the minus strand if its poly-A tail corresponded to a stretch of Ts at the 5′ end of the fragment relative to the reference genome. To filter out cases in which inferred poly-A tails originated from stretches of As or Ts encoded endogenously in the genome, we eliminated from analysis all reads whose stretch of As or Ts contained more than 50% matches to the reference genome. In order to filter out cases of potential oligo-dT mispriming during cDNA synthesis, we also eliminated from analysis all reads that contained 10 or more As in the 20 nucleotides upstream of their transcription termination site. Read mapping statistics can be found in Table S2.

We controlled for read abundance biases due to differing GC content as follows. For each lane of sequencing, we grouped sets of overlapping reads and normalized abundance according to GC content of the overlapping region using full-quantile normalization as implemented in the package EDASeq [50]. Normalized abundance was divided by raw abundance to generate a weight that was assigned to every read in the group. These weights were used in place of raw read counts in all downstream analyses. All expression data are available through the Gene Expression Omnibus under identification number GSE38875.

### Transcript annotation

Coordinates of orthologous open reading frames (ORFs) in each genome were taken from http://www.saccharomycessensustricto.org. These ORF boundaries in *S. cerevisiae* differed, in some cases, from ORF definitions in the *Saccharomyces* Genome Database [51, SGD, using the definitions from December 22, 2007]; genes for which the two sets of definitions did not overlap were discarded. For cases where the definitions overlapped but differed by more than ten base pairs at either end, we used the boundaries defined by SGD and adjusted ortholog boundaries in other species accordingly after performing local multiple alignment [52] of the orthologous regions and flanking sequences as defined by [27].

For most genomic loci, each sense transcript feature was defined as the region from 50 bp upstream to 500 bp downstream of its respective ORF. If sequence within this window for a given target ORF overlapped with the boundaries of an adjacent gene or known non-coding RNA on the same strand, the sense feature boundaries of the target were trimmed to eliminate the overlap. For tandem gene pairs, the 3′ boundary of the upstream gene sense feature was set to 500 bp past the coding stop or the coding start of the downstream gene sense feature, whichever was closer; the 5′ boundary of the downstream gene sense feature was set to 50 bp upstream of its coding start or the 3′ end of the upstream gene sense feature, whichever was closer.

We tabulated the GC-normalized expression counts (see above) that mapped to each transcript feature for each RNA-seq sample. Given the full set of such counts across all features and all samples, we then applied the upper-quartile between-lane normalization method implemented in EDASeq [50]. The normalized counts from this latter step for a given species were averaged across all biological replicates to yield a final expression level for the feature, which we then 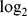 transformed and used in all analysis in this work.

### Yeast pathways

We downloaded the list of genes associated with each Gene Ontology process term from the *Saccharomyces* Genome Database and filtered for terms containing at least 10 genes. The resulting set comprised 333 terms.

### Visualizing distributions of interspecific expression variation

For visual inspection of expression differences between species in Figure 4, we normalized experimentally measured data by branch lengths ascertained from genome sequence as follows. If expression evolution follows the same Gaussian-based model on all lineages of the yeast phylogeny, when the expression level of gene 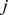 in taxon 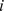 is compared to that in taxon 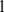 used as a reference, the marginal distribution 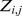 (the difference in expression between taxon 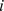 and taxon 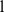 at gene 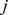) is distributed according to a univariate analog of equation (3). In this case, dividing 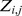 by the absolute branch length according to DNA sequence between taxon 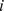 and taxon 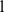 eliminates the dependence of the distribution on the total divergence time between taxa, and the density of this normalized quantity will be the same for all species comparisons. In the case of lineage-specific shifts in evolutionary rate or universal selective constraint, one or more taxa will exhibit distinct densities of the normalized expression divergence measure. Thus, we generated each distribution in Figure 4 by tabulating the log fold-change in expression between the indicated species and *S. cerevisiae*, and then dividing this quantity by the divergence time between the indicated species and *S. cerevisiae* according to the genome tree. After this normalization, if a pathway has been subject to accelerated regulatory evolution in one lineage, the distribution of expression log fold-changes corresponding to the species at the tip of that lineage will be wider than expected based on the length of the branch from DNA sequence, and hence it will stand out against the other distributions when plotted as in Figure 4; likewise, constraint on expression evolution of a pathway in a particular species will manifest as a narrower distribution for that species. In the case of a pathway subject to the same degree of regulatory constraint on all branches of the yeast phylogeny, branch lengths ascertained from genome sequence will be large relative to the modest expression divergence, with the most dramatic disparity manifesting when divergent species are compared, yielding the narrowest distribution of normalized expression levels. When visualized as in Figure 4, the width of the distribution of log fold-changes across genes of the pathway in a given species will thus be inversely proportional to the species distance from *S. cerevisiae*, with the narrowest distribution for *S. bayanus* and the widest for *S. paradoxus*.

## Supporting Information

Figure S1 **Phylogenetic inference of the evolutionary history of yeast pathway regulation under a Brownian motion model with equal rates on each branch of the tree.** Data are as in Figure 1a of the main text except that expression data were simulated under a model in which no yeast lineage was subject to a change in evolutionary rate.
(EPS)

Figure S2
**Phylogenetic inference of the evolutionary history of yeast pathway regulation under a model with a rate shift on the subtree leading to** ***S. paradoxus*** **and** ***S. cerevisiae*** **.** (a), Data are as in Figure 1a of the main text, except that expression measurements were simulated under a Brownian motion model in which the rate of regulatory evolution for each gene was drawn from an inverse-gamma distribution with 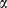 = 3, 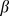 = 2 and, for the subtree leading to *S. paradoxus* and *S. cerevisiae*, increased by a factor of 5. (b), Data are as in Figure 1b of the main text, except that expression measurements were simulated under a Brownian motion model in which the rate of regulatory evolution for each gene was drawn from an inverse-gamma distribution with 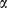 = 3, 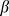 = 2 and, for the subtree leading to *S. paradoxus* and *S. cerevisiae*, increased by the factor indicated on the 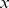 axis.(EPS)

Figure S3
**Phylogenetic inference of the evolutionary history of yeast pathway regulation under a model with a rate shift on the branch leading to** ***S. cerevisiae*** **.** (a), Data are as in Figure 1a of the main text, except that expression measurements were simulated under a Brownian motion model in which the rate of regulatory evolution for each gene was drawn from an inverse-gamma distribution with 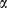 = 3, 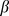 = 2 and, for the branch leading to *S. cerevisiae*, increased by a factor of 5. (b), Data are as in Figure 1b of the main text, except that expression measurements were simulated under a Brownian motion model in which the rate of regulatory evolution for each gene was drawn from an inverse-gamma distribution with 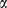 = 3, 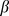 = 2 and, for the branch leading to *S. cerevisiae*, increased by the factor indicated on the 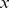 axis.(EPS)

Figure S4
**Phylogenetic inference of the evolutionary history of yeast pathway regulation under a model with a rate shift on the branch leading to** ***S. mikatae*** **.** (a), Data are as in Figure 1a of the main text, except that expression measurements were simulated under a Brownian motion model in which the rate of regulatory evolution for each gene was drawn from an inverse-gamma distribution with 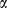 = 3, 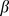 = 2 and, for the branch leading to *S. mikatae*, increased by a factor of 5. (b), Data are as in Figure 1b of the main text, except that expression measurements were simulated under a Brownian motion model in which the rate of regulatory evolution for each gene was drawn from an inverse-gamma distribution with 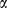 = 3, 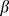 = 2 and, for the branch leading to *S. mikatae*, increased by the factor indicated on the 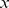 axis.(EPS)

Figure S5
**Phylogenetic inference of the evolutionary history of yeast pathway regulation under a model with a rate shift on the branch leading to** ***S. bayanus*** **.** (a), Data are as in Figure 1a of the main text, except that expression measurements were simulated under a Brownian motion model in which the rate of regulatory evolution for each gene was drawn from an inverse-gamma distribution with 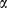 = 3, 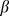 = 2 and, for the branch leading to *S. bayanus*, increased by a factor of 5. (b), Data are as in Figure 1b of the main text, except that expression measurements were simulated under a Brownian motion model in which the rate of regulatory evolution for each gene was drawn from an inverse-gamma distribution with 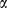 = 3, 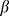 = 2 and, for the branch leading to *S. bayanus*, increased by the factor indicated on the 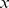 axis.(EPS)

Figure S6
**Relationship between inferred values of parameters in phylogenetic reconstruction of the evolutionary history of yeast pathway regulation, under an Ornstein-Uhlenbeck model.** In the main plot, each data point reports the results of inference of the evolutionary history of regulation of a yeast pathway of size 100: expression data were simulated under an Ornstein-Uhlenbeck (OU) model in which the rates of regulatory evolution of pathway genes were drawn from an inverse-gamma distribution with 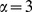 and 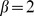 and the OU constraint parameter 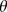 was set to 10, after which parameter values for an OU model were optimized against the simulated expression data. For histograms at top and left, the independent variable is shared with the axis of the main plot and reports the indicated parameter value, and the dependent variable reports the proportion of simulated data sets in which the corresponding value was inferred. Note that inferences from most simulated data sets accurately estimate 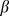 and 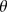, but for a few data sets, large parameter values are inferred.(EPS)

Figure S7
**Mean-centering pathway expression levels in each species corrects for spurious inference of non-neutral regulatory evolution arising from gene co-regulation.** Each trace reports the results of inference of the evolutionary history of regulation of a yeast pathway of size 100, from expression data simulated under a Brownian motion model in which evolutionary rates were the same on all branches of the yeast phylogeny, and pathway genes were correlated with one another with respect to expression throughout the phylogeny. Each line style reports one scheme for normalization of simulated expression data before evolutionary inference: expression measurements were analyzed as is (Uncentered), or the distribution of expression across pathway genes for each species in turn was normalized to have a mean of 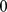 (Centered). The 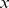 axis reports the value of the correlation coefficient between genes in the group, and the 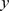 axis reports the fraction of 500 simulations that resulted in a model other than the Brownian motion equal-rates model having an Akaike weight greater than 0.8.(EPS)

Figure S8
**Heterogeneity in the mode of regulatory evolution across the genes of a pathway has little impact on inference of evolutionary histories from expression data.** Each trace reports the results of inference of the evolutionary history of regulation of a yeast pathway of size 100, from expression data simulated under a Brownian motion model in which the rate of regulatory evolution for each gene was drawn from an inverse-gamma distribution with 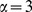, 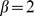 and, for the branch leading to *S. paradoxus*, increased by a factor of 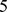 for a subset of pathway genes. The 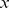 axis reports the fraction of genes in the group without a rate shift, and the 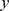 axis reports the average Akaike weight assigned to each model. Line styles are as in Figure 1a of the main text.(EPS)

Table S1 **Strains used in this work.**
(XLSX)

Table S2 **Read mapping statistics from yeast RNA-seq.** Each set of rows reports the mapping statistics for reads from RNA-seq libraries used for a comparison of two yeast species. For a given set, in row headings, numerals indicate biological replicates, single species names indicate homozygotes, and species name pairs separated by a slash indicate diploid interspecies hybrids. Each row reports results from one library. Total reads, the full set of reads sequenced. Have polyT, the number of reads containing at least two consecutive Ts at only one end. Uniquely mapped, the number of reads mapping uniquely, with no mismatches, to the concatenated genomes of the two species of the set. Passed through filters, the number of reads whose poly-A tails were unlikely to have originated from oligo-dT mispriming to A-rich regions of the genome; see Methods.
(XLSX)

Table S3
**Fitted models of** 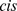 **-regulatory evolution in yeast pathways.** Data are as in Table 1 of the main text except that results for all pathways are shown.(XLSX)

Table S4 **Fitted models of species regulatory evolution in yeast pathways.** Data are as in Table 2 of the main text except that results for all pathways are shown.
(XLSX)
